# Associations between past trauma, current social support, and loneliness in incarcerated populations

**DOI:** 10.1186/2194-7899-2-7

**Published:** 2014-04-01

**Authors:** Jennifer C Kao, Adam Chuong, Madhavi K Reddy, Robyn L Gobin, Caron Zlotnick, Jennifer E Johnson

**Affiliations:** 1 Brown University – Department of Psychiatry and Human Behavior, 700 Butler Drive, Providence, RI 02906 USA; 2 Brown University – Department of Psychiatry and Human Behavior, Psychosocial Research Program, 345 Blackstone Blvd, Providence, RI 02906 USA; 3 VA San Diego Healthcare System, La Jolla Village Drive, San Diego, CA 92161 USA; 4 Butler Hospital, 345 Blackstone Blvd, Providence, RI 02906 USA

**Keywords:** Trauma, Abuse, Social support, Incarceration, Prisoners

## Abstract

**Background:**

Prisoners are a vulnerable population with higher rates of trauma than community populations. Social support is important for both in–prison adjustment and post-release community re-entry. Loneliness, a related construct to social support, has been found to be associated with elevated rates of suicidal ideation and behavior, depression, and hopelessness in incarcerated populations.

**Methods:**

This study explored the relationship of past physical, sexual, and crime-related trauma to current perceived social support and loneliness in a mixed-gender sample of 235 depressed prisoners enrolled in depression treatment studies.

**Results:**

History of any trauma, regardless of type, as well as physical, sexual, and crime-related traumas were associated with lower current perceived social support scores (controlling for gender). Past sexual trauma and crime-related trauma were associated with higher levels of loneliness (controlling for gender), though history of any trauma and physical trauma was not.

**Conclusion:**

Findings suggest that both overall trauma and specific types of trauma are linked to perceived social support and loneliness among incarcerated persons with major depressive disorder. This is the first study, to the authors’ knowledge, that specifically observed a relationship between past trauma and current social support and loneliness in prison populations. Many intervention programs for prisoners encourage them to develop non-criminogenic and substance-free social networks. These programs should be aware that individuals who have experienced interpersonal trauma may have additional interpersonal challenges, and may need strategies to address interpersonal deficits related to trauma exposure.

**Electronic supplementary material:**

The online version of this article (doi:10.1186/2194-7899-2-7) contains supplementary material, which is available to authorized users.

## Background

Prisoners experience interpersonal trauma at higher frequencies than individuals in non-incarcerated populations (Browne et al. 1999; Bosgelmez et al. 2010). By interpersonal trauma, we are referring to events such as interpersonal violence or abuse, including physical or sexual assault/abuse and being the victim of interpersonal crime. Several studies with prisoners have assessed trauma history prior to incarceration. In studies of prisoners, lifetime rates of interpersonal trauma ranged from 90% (B. L. Green et al. 2005) to 94% (Browne et al. 1999) in women and from 54.3% (Carlson et al. 2010) to 95% (Komarovskaya et al. 2011) in men. In both the general population and in prisoners, a trauma history has been associated with a range of difficulties such as depression, personality disorders, PTSD, substance abuse, anxiety, and suicide risk (Blaauw et al. 2002; Esposito and Clum 2002; Mullings et al. 2004; Nishith et al. 2000; Wise et al. 2001; Zweig et al. 2012). In addition, trauma exposure has often been linked, both directly and indirectly, to both men and women’s involvement in the criminal justice system (Asberg and Renk 2013; DeHart 2008; Curtis et al. 2001; Thornberry et al. 2010). Since trauma exposure is very common among incarcerated populations and leads to a wide range of negative outcomes, it is important to further explore and understand the potential effects of past trauma on these populations.

Interpersonal trauma may lead to interpersonal deficits as disclosure of trauma may lead to negative reactions from those in one’s social network, the source of interpersonal violence may be people who also provide social support, and trauma may have a negative influence on one’s level of trust and boundaries within interpersonal relationships (Asberg and Renk 2013; Janoff-Bulman 2010). It is therefore important to understand how traumatic experiences, common among incarcerated populations, impact their interpersonal relationships. Past interpersonal trauma has been consistently linked to lower levels of social support in the general population (Golding et al. 2002; Muller et al. 2008; Pepin and Banyard 2006; Thompson et al. 2000). However, it is unclear whether a relationship between past trauma and social support exists within incarcerated populations. If it did, this would be of concern because social support (i.e., “perceived or actual instrumental and/or expressive provisions supplied by the community, social networks, and confiding partners” (Lin 1986)) serves as an important protective factor for prisoners both during incarceration and at re-entry. Low perceived social support, referring to support from both inside and outside the prison (“everyone you know”), is associated with more suicide attempts in prison (Meltzer et al. 2003), difficulties in community re-entry, substance use relapse and recidivism (Parsons and Warner-Robbins 2002; Liau et al. 2004; Benda 2005). Among both incarcerated and non-incarcerated individuals, social support, specifically perceived emotional and practical support and number of people perceived as supportive, has also been found to be protective against various physical and psychological health outcomes such as depression, anxiety, and hopelessness (Johnson et al. 2011; Biggam and Power 1997; Bradley and Cartwright 2002).

Although the importance of social support for prisoners is well-recognized, inmates often receive inadequate social support (Asberg and Renk 2012). For example, inmates’ contact with their closest friends and family is usually rationed and sparse (Biggam and Power 1997). One of the only studies, to the authors’ knowledge, looking at trauma and social support in prisoners is a small study (Asberg and Renk 2012) comparing perceived social support between women inmates with childhood abuse (n = 24) and those without (n = 15); they found no significant difference between the two groups. However, this lack of significance may have been due to the study’s small sample size. Given the important consequences of social support for prisoners, more research is needed to understand how factors such as past trauma may influence social support.

Loneliness is a construct related to social support which refers to one’s subjective feelings of loneliness and feelings of social isolation (operationalized in this study by the UCLA Loneliness Scale, which includes questions such as how often one feels alone, feels that no one understands, or feels unable to reach out or communicate with others; UCLA-LS; Russell 1996). In incarcerated populations, subjective feelings of loneliness have been found to be associated with elevated rates of suicidal ideation and behavior, depression, and hopelessness (Brown and Day 2008). In fact, Brown and Day’s (2008) study of 60 inmates found that those who reported higher levels of loneliness also scored higher on indicators of suicidal behavior. A study by Bonner and Rich (1990) with 146 inmates found a link between loneliness and suicidal ideation. Experiences of interpersonal trauma may lead to negative internal reactions that influence feelings of loneliness, such as feelings self-blame (Oaksford and Frude 2004) and feeling detached or isolated from others (DSM-5; American Psychiatric Association 2013). Loneliness has also been found to be associated with history of trauma and abuse in the general population (Rokach 2007; Gibson and Hartshorne 1996; Prinstein et al. 2001).

Virtually no studies have examined the association between past trauma and loneliness among incarcerated populations. Two studies examined within-prison bullying in male prisoners and found that victims of such bullying experienced higher loneliness, though these investigations did not examine trauma before incarceration (Ireland and Power 2004; Ireland and Qualter 2008). As loneliness has been linked to negative mental health outcomes in incarcerated populations and history of trauma has been seen to affect loneliness in non-incarcerated populations, it is important to understand the possible relationships between history of trauma and loneliness in prisoners.

This study examines past interpersonal trauma in relation to current perceived social support and to loneliness in a convenience sample of male and female prisoners (97% of whom reported problematic substance use) with a major depressive disorder who volunteered for a depression treatment research study. Because some studies of non-incarcerated samples have found that sexual and physical trauma may have differential effects on victims (Golding et al. 2002; Muller et al. 2008; Meyerson et al. 2002), we also examine the associations between trauma and social support/loneliness separately for sexual trauma, physical trauma, and crime-related trauma (defined as robberies and break-ins).

Both depression and substance use have been associated with lower perceived social support and with trauma history in incarcerated (Borelli et al. 2010; Johnson 2004; Vranceanu et al. 2007) and community populations (Galea et al. 2004; Kendler et al. 2004; Peirce et al. 2000; Simpson and Miller 2002; Stice et al. 2004). Because these disorders were virtually constant in our sample (i.e., all participants met criteria for MDD and 97% met diagnosis for SUD or reported problematic substance use), these conditions were not accounted for in our analyses but are also unlikely to serve as confounders of the relationship between trauma and social support. The primary caveat with the use of this convenience sample is that social support may be lower and rates of trauma higher than in other incarcerated samples.

We hypothesize that: 1) the presence of any past trauma will be associated with lower levels of perceived social support and higher self-reported loneliness; and 2) physical trauma, sexual trauma, and crime-related trauma will each be associated with lower levels of current perceived social support and higher self-reported loneliness. If past trauma history is found to be associated with impairments in current support networks and relationships, this has implications for a broad range of interventions that seek to help prisoners engage with more positive (i.e., non-criminogenic, non-substance involved) social networks, and those, such as case management, that seek to engage them in community services. For example, these interventions and services may be more effective or appropriate if they account for or address long-term deficits in interpersonal functioning related to traumatic experiences.

## Methods

Data for this secondary analysis were drawn from baseline interviews (n = 235) of three previous and one ongoing study of variants of interpersonal psychotherapy (IPT) for major depressive disorder (MDD) among women and men in prison (Johnson and Zlotnick 2008, 2012; Johnson et al. in press). All relevant measures were administered in each study, with the exception of loneliness, which was only administered in the ongoing study. Therefore, analyses predicting loneliness use a subset of the sample (n = 149).

### Participants

For all parent studies, potential participants were recruited through flyers or announcements made in the prison residential areas describing the depression treatment studies. Participants privately volunteered for the initial assessment. Study staff conducted screening and consent procedures individually and privately. Staff read the consent form out loud and emphasized the voluntary nature of study participation. There were no legal incentives for participation. Participants received compensation for completing follow-up assessments (amounts varied across studies from U.S. $10 to U.S. $30 per assessment). The studies followed ethical guidelines for research with prisoners under institutional ethics review board approval.^a^

Studies shared a majority of inclusion criteria. All participants met criteria for current primary (non-substance-induced) MDD as determined by the Structural Clinical Interview for the DSM-IV Axis I Disorders (SCID-I; First et al. 1996) after at least 4 weeks of incarceration. Participants who met SCID-I lifetime criteria for bipolar disorder, psychotic disorder, or were at imminent suicidal risk were excluded. Differences in inclusion criteria across studies are discussed below.

Studies 1–3. Participants included sentenced female prisoners (n = 26, Johnson and Zlotnick 2008; n = 38 - Johnson and Zlotnick 2012; n = 22, Johnson et al. in press) from minimum and medium security state prisons in Rhode Island and Massachusetts. Because these studies examined the effects of depression treatment on post-release substance use, participants met criteria for substance use disorder (SUD; abuse or dependence on alcohol or drugs) one month prior to incarceration, as determined by the SCID, in addition to current primary (non-substance induced) MDD. Furthermore, participants were required to be 10 to 24 weeks away from prison release.

Study 4 enrolled men (n = 83) and women (n = 66) in minimum and medium security prisons in Rhode Island and Massachusetts. Because the purpose of this study was to evaluate effectiveness of prison depression treatment on in-prison outcomes only, there were no SUD inclusion/exclusion criteria. Participants were required to expect to be at their current facility for at least 6 months, so study follow-ups could take place in prison. Baseline interviews, which took place in prison and which provide the data for the current analysis, were very similar between Studies 1–3 and Study 4.

### Assessments

#### Trauma

Presence of interpersonal trauma was measured by a shortened version of the Trauma History Questionnaire (THQ; Green 1996), a standard measure of trauma exposure, consisting of 12 items measuring the presence and frequency of lifetime interpersonal trauma. The test-retest reliability of the THQ has been found to be moderate to high for different traumas (e.g., attacked with a weapon, robbed, unwanted sex), with test-retest correlations ranging from .54 to .92 (Green 1996). The scale can be further broken down into three forms of trauma: physical, sexual, and crime-related. The shortened version of the THQ utilized in this study included all items from the physical and sexual trauma subscales. Specifically, sexual trauma was assessed by questions asking about unwanted sex, inappropriate touching, and attempts to engage in unwanted sexual contact. Physical trauma was assessed by questions about assault with a weapon, assault without a weapon that resulted in injury, physical assault by a family member, and having objects thrown at oneself. Two items were removed from the 4-item crime-related trauma subscale for this shortened version, as they were crime-related trauma that did not have direct interpersonal contact (e.g., has anyone ever attempted to or succeeded in breaking into your home when you were not there?”). Crime-related trauma was assessed by asking about muggings and about break-ins while one was at home.

#### Social support

Participants completed the Multidimensional Scale of Perceived Social Support (MSPSS, Zimet et al. 1988), a 12 item measure of perceived social support. The MSPSS asks questions such as, “I can count on my friends when things go wrong” and includes 3 subscales for each source of support (i.e., Family, Friends, or Significant Other). Each group consists of four items rated on a 7-point Likert scale ranging from 1 - *very strongly disagree* to 7 - *very strongly agree*. The MSPSS has good internal reliability and construct validity. The Cronbach’s coefficient alpha values in past studies range from .84 to .92 for total scores, .81 to .90 for Familial support, from .90 to .94 for Friend-provided support, and from .83 to .98 for the Significant Other provided support (Zimet et al. 1990).

#### Loneliness

The UCLA Loneliness Scale (UCLA-LS; Russell 1996) is a self-report measure consisting of 10 items rated on a 4-point Likert scale. This scale includes questions such as how often one feels alone, feels that no one understands, or feels unable to reach out or communicate with others (UCLA-LS; Russell 1996). Participants report the frequency of endorsing each item as *often, sometimes, rarely,* or *never.* A total score is calculated with higher scores indicating greater feelings of loneliness. The scale was developed from statements that 20 psychologists used to describe loneliness, which were also correlated with responses to other self-reports of current loneliness. The UCLA-LS has good test-retest (r = .73 over a 1-year period) and internal reliability (Cronbach’s alpha ranging from .89 to .94; Russell, 1996). Additionally, it has been shown to have construct validity with correlations between loneliness and measures of health and well-being (Russell 1996).

### Analyses

The primary data analyses used combined baseline data from four parent studies. Comparisons of the samples were conducted to check the validity of aggregating the data. There were no significant differences in depressive symptoms severity as measured by the 17-item Hamilton Rating Scale for Depression (Hamilton 1960), perceived social support (MSPSS) or trauma exposure (y/n as measured by the THQ) between Studies 1–3 and Study 4.

To test the association between past trauma and current social support, we conducted four separate linear regressions using history of any trauma and of the three forms of trauma in the THQ (i.e., any trauma, sexual trauma, physical trauma, and crime-related trauma) as the independent variables and MSPSS total score as the dependent variable. To test the association between past trauma and current loneliness, we conducted four similar linear regression analyses with loneliness as the dependent variable. Because gender is known to be related to both trauma incidence and social support in incarcerated populations (Peters et al. 1997; Jiang and Winfree 2006), gender was included as a covariate in all analyses. Primary analyses were conducted in the complete sample. Secondary analyses reported results separately for men and women when sufficient variation in trauma variables was present.

#### Power considerations

With 235 participants and trauma rates as reported (83.4% for physical trauma, 55.7% for sexual trauma, 56.6% for crime-related trauma and 88.9% for any trauma), the study has 80% power to detect an effect size (Cohen’s d) of d = 0.49 for physical trauma, 0.37 for sexual trauma, 0.37 for crime-related trauma, and 0.59 for any trauma predicting social support. In the subset of the sample (n = 149) for which loneliness data are available, trauma rates were (85.9%, 50.3%, 58.4%, and 91.3%), giving the study 80% power to detect Cohen’s d of 0.66 for physical trauma, 0.47 for sexual trauma, 0.47 for crime-related trauma, and 0.83 for any trauma in predicting loneliness. Therefore, the study has better power to detect effects of sexual and crime-related trauma on social support and loneliness, and better power for analyses predicting social support than for those predicting loneliness. Because power varies across tests using different kinds of loneliness, results also report effect sizes.

## Results

### Participants

Descriptive data for the sample are shown in Table 1. Of the total sample of 235 participants, 37 (15.7%) were African-American/Black, 2 (0.9%) Asian, 3 (1.3%) Native American/Alaskan Native, 162 (68.9%) Caucasian, and 31 (13.2%) “Other”. Participants’ mean age was 38.0 (SD = 10.2). 88.9% of participants reported experiencing at least one past interpersonal trauma of some kind, 55.7% reported past sexual trauma, 83.4% physical trauma, and 56.6% crime-related trauma. In women, 86.5% reported any trauma, 67.4% sexual trauma, 79.7% physical trauma, and 53.5% crime-related trauma; in men, 97.6% reported any trauma, 38.6% sexual trauma, 94.0% physical trauma, and 63.9% crime-related trauma. The mean social support (MSPSS) score was 54.4 (SD = 17.5) for all participants, 56.8 (17.1) for women, and 50.0 (17.5) for men. The mean loneliness (UCLA Loneliness) score was 19.2 (SD = 6.03) for all participants, 18.2 (17.1) in women, and 20.0 (5.83) in men. Table 2 shows correlations among variables used in linear regression analyses.

**Table 1:** **Sample Characteristics (n = 235)**

Demographics	
Age [M (SD)]	38.0 (10.2)
Gender	
Female	64.7%
Male	35.3%
Ethnicity	
Hispanic	18.7%
Not Hispanic	81.3%
Race	
African-American	15.3%
Asian	0.90%
Native American/Alaskan Native	1.70%
White	70.2%
Other	11.9%
Unmarried	83.0%
Annual legal income < $10,000 USD prior to incarceration	58.4%
Criminal justice characteristics	
Security level	
Minimum security	51.9%
Medium security	48.1%
Median (range) number of past arrests	6 (0–100)
Median (range) months incarcerated to date for current sentence	23 (0–489)
Past trauma exposure	
Any trauma	88.9%
Sexual trauma	55.7%
Physical trauma	83.4%
Crime-related trauma	56.6%
MSPSS perceived social support score [M (SD)]	54.4 (17.5)
UCLA Loneliness Scale score [M (SD)]	19.2 (6.0)

**Table 2:** **Spearman correlations among study variables (n = 235)**

	Social support	Loneliness	Presence of any trauma	Presence of physical trauma	Presence of sexual trauma	Presence of crime-related trauma	Gender = Female
Social support	**_______**						
Loneliness	-.494**	_______					
Presence of any trauma	-.175**	.131	_______				
Presence of physical trauma	-.150*	.087	.768**	_______			
Presence of sexual trauma	-.084	.177*	.374**	.170*	_______		
Presence of crime-related trauma	-.253**	.223**	.378**	.297**	.342**	_______	
Gender = Female	.180**	-.142	-.181**	-.191**	.279**	-.102	_______

* p < .05.

** p < .01.

### Trauma predicting social support

Controlling for gender, past history of any trauma (regardless of type), sexual trauma, physical trauma, and crime-related trauma were all individually associated with lower perceived social support in the aggregate sample (see Table 3). When men and women were analyzed separately, effects were clearer among women than among men (see Table 4), although post hoc tests of interactions between gender and trauma type in predicting social support were not significant. Effect sizes of trauma types on social support were mostly in the medium range: sexual trauma was associated with a d = 0.44 less social support among women and d = 0.02 among men, and crime-related trauma with d = 0.57 less social support among women and d = 0.40 less social support among men. Effect sizes for any trauma and physical trauma among women were d = 0.57 and d = 0.47, respectively; too many men reported these experiences to make comparisons among men meaningful.

**Table 3:** **Associations between current social support and history of trauma by type, with gender as a covariate**

	B	SE B	β
Any Trauma (n = 231)	−9.520	3.902	-.159*
Gender = Female	5.827	2.387	.159*
Physical Trauma (n = 231)	−6.702	3.211	-.137*
Gender = Female	5.930	2.400	.162*
Sexual Trauma (n = 230)	−4.906	2.387	-.138*
Gender = Female	8.228	2.460	.225**
Crime-Related Trauma (n = 233)	−8.758	2.225	-.248**
Gender = Female	5.970	2.299	.164**

* p < .05.

** p < .01.

**Table 4:** **Associations between current social support and history of trauma by type and gender**

	Male (n = 83)			Female (n = 150^b^)		
	B	SE B	β	B	SE B	β
Any trauma^a^	**_______**	**_______**	**_______**	−9.791	4.074	-.195*
Physical trauma^a^	**_______**	**_______**	**_______**	−8.070	3.469	-.189**
Sexual trauma	-.434	3.961	-.012	−7.626	2.979	-.208*
Crime-related trauma	−6.867	3.940	-.190	−9.729	2.694	-.285**

* p < .05.

** p < .01.

a. Regressions predicting social support from any trauma and physical trauma were not conducted for men because only 2 men reporting not having experienced any trauma, and only 5 men reported not having experienced physical trauma.

b. For females, n = 147 (sexual trauma), 148 (physical trauma), 150 (crime-related trauma), 148 (any trauma).

### Trauma predicting loneliness

Controlling for gender, past history of sexual trauma and crime-related trauma were individually associated with more loneliness in the aggregate sample (see Table 5),. When men and women were analyzed separately, effects seemed roughly similar across genders (see Table 6), and post hoc tests of interactions between gender and trauma type in predicting loneliness were non-significant. Effects for significant predictors (sexual and crime-related trauma) on loneliness were medium-small to medium-large. History of sexual trauma was associated with a d = 0.66 more loneliness among women and d = 0.32 among men. History of crime-related trauma was associated with a d = 0.40 more loneliness among women and d = 0.43 among men. Effect-sizes for non-significant predictors of loneliness (any trauma and physical trauma) among women were d = .31 and d = 0.13, respectively; too many men reported these experiences to make comparisons among men meaningful.

**Table 5:** **Associations between current loneliness and history of trauma by type, with gender as a covariate (n = 149)**

	B	SE B	β
Any Trauma	2.465	1.790	.116
Gender = Female	−1.373	1.017	-.114
Physical Trauma	1.034	1.462	.060
Gender = Female	−1.536	1.024	-.127
Sexual Trauma	2.822	.994	.235*
Gender = Female	−2.475	1.00	-.205*
Crime-Related Trauma	2.492	.985	.204*
Gender = Female	−1.417	.977	-.117

* p < .05.

** p < .01.

**Table 6:** **Associations between current loneliness and history of trauma by type and gender**

	Male (***n*** = 83)			Female (***n*** = 66)		
	B	SE B	β	B	SE B	β
Any trauma^a^	**_______**	**_______**	**_______**	1.909	2.043	.116
Physical trauma^a^	**_______**	**_______**	**_______**	.795	1.786	.056
Sexual trauma	1.858	1.307	.156	4.086	1.525	.318*
Crime-related trauma	2.535	1.310	.210	2.443	1.503	.199

* p < .05.

** p < .01.

a. Regressions predicting loneliness from any trauma and physical trauma were not conducted for men because only 2 men reporting not having experienced any trauma, and only 5 men reported not having experienced physical trauma.

## Discussion

This study examined trauma history in relation to perceived social support and loneliness in a sample of incarcerated women and men with major depressive disorder. A history of any trauma (regardless of type), physical trauma, sexual trauma, and crime-related trauma were all associated with current lower social support In our sample, past sexual trauma and crime-related trauma only were significantly associated with higher levels of current self-reported loneliness, though physical trauma and any trauma were not. This is the first study, to the authors’ knowledge, that specifically observed a relationship between past trauma and current social support and loneliness in prison populations. Findings contribute to the general literature by examining the effects of different kinds of trauma on interpersonal functioning among women and among men.

Past exposure to any trauma was associated with lower social support, as hypothesized and in line with the literature in non-incarcerated populations. This suggests that exposure to trauma may affect prisoners similarly to non-incarcerated populations. This makes sense as reasons cited in studies with non-incarcerated populations of why trauma may affect social support is similarly applicable to incarcerated populations: others’ negative reactions to trauma disclosure, negative influence on the victim’s level of trust and security in relationships, and lack of access to safe social support interpersonal trauma is commonly experienced in families and friendships (Asberg and Renk 2013; Janoff-Bulman 1992; Onyskiw 2001). History of any trauma was, however, not with loneliness in our results. Because the vast majority (91.3%) of the 149 participants with loneliness data reported having experienced “any trauma,” power for this variable was poor in our sample; further research is needed.

When specific types of trauma are considered, our results suggesting an association between past sexual trauma and both perceived social support and loneliness in prisoners, are in line with similar studies in community populations (Golding et al. 2002; Gibson and Hartshorne 1996). Golding, Wilsnack, and Cooper (2002) found, using data from six non-incarcerated samples, that individuals with sexual trauma reported lower current emotional support, and Gibson and Hartshorne (1996) found that victims of sexual abuse experienced more loneliness in their mixed sample of female university students and female clients at treatment centers. Though we were unable to explore the reasons for the association between trauma and loneliness/social support among incarcerated populations found in the current study, the wider literature on sexual trauma provides possible explanations. For example, several studies examining coping in adults with histories of childhood sexual abuse have found that survivors engage in strategies such as social withdrawal and self-isolation (Griffing et al. 2006; Futa et al. 2003) and are less likely to seek out social support. Victims of sexual trauma may also engage in behaviors that indirectly impact their social support and levels of loneliness such as acting out sexually and aggressively (Filipas and Ullman 2006), emotional suppression (Brand and Alexander 2003), self-blame (DiLillo et al. 1994; Futa et al. 2003; Oaksford and Frude 2004), addictive behaviors or substance use (Brand et al. 1997; Filipas and Ullman 2006), and decreased self-esteem and life satisfaction (Fergusson et al. 2008).

Physical trauma was related to social support but not to loneliness in the current study. Several studies in non-incarcerated populations have also found associations between physical trauma and perceived social support (Elliott and Briere 1992; Muller et al. 2008; Runtz and Schallow 1997). Our findings about the lack of association between physical trauma and loneliness should be considered preliminary, because the study only had power to detect large effects for this association (due to a lower sample size for loneliness than for social support and the fact that 6 of 7 people in our sample had experienced physical trauma). Therefore, our results primarily suggest that the association between history of physical trauma and current loneliness is not a large effect.

Crime-related trauma, defined in this study as robberies and break-ins, has not been explored much in either the community or incarcerated samples in relationship to social support or to loneliness. However, experience of crime-related trauma have been linked to other negative sequelae, such as the development of acute stress disorder and depressive symptomatology, in the general population (Elklit 2002; Hochstetler et al. 2010). Our results suggest that the experience of past crime-related trauma is associated with lower current social support and with higher loneliness. Crime-related trauma may impact perceived social support through crimes’ impact on the living environment and neighborhood of the individual. It may be harder to establish social support networks in environments with high rates of robberies and break-ins, or when one is fearful of others who may perpetrate a crime, which in turn may negatively affect perceived social support and loneliness. Elliott and colleagues’ (2005) study on adolescents, which found that those from riskier neighborhoods were more socially isolated, provides some evidence for this explanation. However, low social support may also predispose individuals to being the target of crime and could be a possible explanation for the association if current social support is consistent with social support at the time of exposure to crime-related trauma.

Tests of the interaction between types of trauma and gender in predicting social support were not significant, meaning that our results do *not* provide evidence that the association between trauma history and current social support/loneliness is different for women than it is for men. However, there were fewer men than there were women in the sample, and too many of the men had experienced physical or any trauma to make comparisons among men only meaningful for these variables. Further research is needed before conclusions about the role of gender can be drawn.

Associations of trauma with perceived social support and loneliness observed in the current study serve to enhance our understanding of the characteristics and functioning of prisoners and also to bring attention to the disenfranchised situations and vicious cycles of pervasive trauma and inadequate social support that prisoners often face. For example, Asberg and Renk (2013) found that incarcerated women trauma survivors, compared to their college student counterparts, often face less supportive reactions, were more often blamed for their trauma, and were treated more harshly by those around them upon disclosure of trauma. In addition, women with low social support are more likely to be re-victimized (Bender et al. 2003; Bybee and Sullivan 2005). Therefore, inmates’ lack of support in turn may negatively impact posttraumatic adjustment (Robinaugh et al. 2011), which again negatively influences social support.

### Strengths and limitations

This study has several strengths. It included both women and men prisoners, unlike most previous studies looking at social support in incarcerated populations. The sample also included prisoners from across states and security levels. To measure social support, we used a perceived social support scale, which has been more consistently linked to mental health well-being than received social support (Chu et al. 2010; Dolbier and Steinhardt 2000).

This study also has some limitations. This was a convenience sample of prisoners who volunteered to participate in depression treatment studies (all of whom met criteria for major depressive disorder and 97% of whom either met diagnosis for SUD or reported problematic substance use), with a gender distribution not typical of prisoners as a whole, and therefore findings may not extend to other prisoners, even though both substance use and depression are highly prevalent in incarcerated populations (James and Glaze 2006). Secondly, we only examined one kind of social support, perceived emotional support. It is possible that trauma may be differently related to other kinds of social support, such as instrumental social support. Thirdly, the loneliness measure was only administered to half of the sample (n = 149), reducing power for this variable. Finally, the cross-sectional design of the current study limits our ability to determine the direction of the observed associations. Although theory leads us to believe that past trauma precedes low levels of current perceived social support and heightened loneliness, it is also possible that low perceived social support and high rates of loneliness developed prior to trauma exposure. Longitudinal investigations are needed to confirm directionality of these relationships as well as the impact of trauma severity, frequency, and age of onset on perceptions of social support and loneliness.

## Conclusions

Our study highlights the pervasive nature of trauma in the lives of prisoners, and suggests that interpersonal trauma exposure, particularly sexual and crime-related trauma, influence current social support and loneliness among incarcerated individuals. Past studies suggest that exposure to trauma is prevalent among prisoners, that social support is important for within-prison and post-release re-entry functioning, and also that social support and loneliness are linked to negative health outcomes. In this study, we found that various kinds of interpersonal trauma are associated with lower levels of perceived support and higher levels of loneliness in prisoners.

### Policy implications

These results have implications for interventions and services in prison populations. Assessment of trauma history may help to provide important context for understanding incarcerated individuals’ social and interpersonal challenges. Interventions seek to help prisoners engage with non-criminogenic and non-substance-using social networks and those, such as case management, that seek to engage them in community services may be more effective and appropriate if they assess for trauma history and account for or address long-term deficits in interpersonal functioning related to traumatic experiences. For example, interventionists may be more able to help individuals develop social supports if they acknowledge and are sensitive to the fact that traumatic experiences may have given clients good reasons not to trust others. Intervention strategies may incorporate teaching knowledge about how trauma may affect interpersonal functioning, how to determine if new relationships are safe or warning signs that they are not safe, work on boundaries and incremental trust, or discussions about managing relationships with abusers and finding social support from safe sources (Bedard et al. 2003; Zlotnick et al. 2003). Skills for making appropriate requests to friends and family to get one’s needs met in the aftermath of interpersonal trauma may be particularly important. Case managers should also be aware of potential effects of trauma on individuals’ ability to seek out and engage in in-prison and post-release services. Prison treatment programs with trauma-exposed prisoners may want to include assessment of social support and loneliness, explore the ways in which past interpersonal trauma may be impacting perceived social support and feelings of loneliness, and incorporate strategies, such as trauma-informed interpersonal skills programming, to help ameliorate the poor social support and high levels of loneliness that can be consequences of trauma.

In sum, results suggest that past interpersonal trauma may be part of the challenging social and interpersonal context faced by incarcerated individuals. The context of trauma should be considered when working to address poor social support and high levels of loneliness, which are known to influence prisoner mental and physical health and post-release outcomes. Community studies suggest that, in addition to improving well-being and functioning, increasing social support and decreasing loneliness may also help individuals be more resilient against the effects of future trauma (Hawkley and Cacioppo 2010).

## Authors’ original submitted files for images

Below are the links to the authors’ original submitted files for images. Authors’ original file for figure 1
Authors’ original file for figure 2
Authors’ original file for figure 3
Authors’ original file for figure 4
Authors’ original file for figure 5
Authors’ original file for figure 6

## Abbreviations

IMSPSS: Multidimensional Scale of Perceived Social Support
IPT: interpersonal psychotherapy
MDD: Major Depressive Disorder
PSS: Perceived social support
SUD: Substance Use Disorder
UCLA-L: UCLA Loneliness Scale
THQ: Trauma History Questionnaire.
