# Supplementary material for: Associations between past trauma, current social support, and loneliness in incarcerated populations
Source: Health Justice. 2014 Apr 1;2:7. doi: 10.1186/2194-7899-2-7 (PMC5151509; doi:10.1186/2194-7899-2-7)
Supplement: Supplementary file 1 — Authors’ original file for figure 1 [file 40352_2013_9_MOESM1_ESM.docx]

**Table 1.** Sample Characteristics (n = 235)

| Demographics | |
| --- | --- |
| Age [M (SD)] | 38.0 (10.2) |
| Gender  Female  Male | 64.7%  35.3% |
| Ethnicity |  |
| Hispanic | 18.7 % |
| Not Hispanic | 81.3 % |
| Race |  |
| African-American | 15.3 % |
| Asian | 0.90 % |
| Native American/Alaskan Native | 1.70 % |
| White | 70.2 % |
| Other | 11.9 % |
| Unmarried | 83.0 % |
| Annual legal income < $10,000 USD  prior to incarceration | 58.4 % |
| Criminal justice characteristics |  |
| Security level |  |
| Minimum security | 51.9 % |
| Medium security | 48.1 % |
| Median (range) number of past arrests | 6 (0-100) |
| Median (range) months incarcerated to  date for current sentence | 23 (0 - 489) |
| Past trauma exposure |  |
| Any trauma | 88.9 % |
| Sexual trauma | 55.7 % |
| Physical trauma | 83.4 % |
| Crime-related trauma | 56.6 % |
|  |  |
| MSPSS perceived social support score [M (SD)] | 54.4 (17.5) |
| UCLA Loneliness Scale score [M (SD)] | 19.2 (6.0) |
