# Supplementary material for: Associations between past trauma, current social support, and loneliness in incarcerated populations
Source: Health Justice. 2014 Apr 1;2:7. doi: 10.1186/2194-7899-2-7 (PMC5151509; doi:10.1186/2194-7899-2-7)
Supplement: Supplementary file 2 — Authors’ original file for figure 2 [file 40352_2013_9_MOESM2_ESM.docx]

**Table 2.** Spearman correlations among study variables (n = 235).

|  | Social support | Loneliness | Presence of any trauma | Presence of physical trauma | Presence of sexual trauma | Presence of crime-related trauma | Gender = Female |
| --- | --- | --- | --- | --- | --- | --- | --- |
| Social support |  |  |  |  |  |  |  |
| Loneliness | -.494** |  |  |  |  |  |  |
| Presence of any trauma | -.175** | .131 |  |  |  |  |  |
| Presence of physical trauma | -.150* | .087 | .768** |  |  |  |  |
| Presence of sexual trauma | -.084 | .177* | .374** | .170* |  |  |  |
| Presence of crime-related trauma | -.253** | .223** | .378** | .297** | .342** |  |  |
| Gender = Female | .180** | -.142 | -.181** | -.191** | .279** | -.102 |  |

* p < .05

** p < .01
