# Supplementary material for: Associations between past trauma, current social support, and loneliness in incarcerated populations
Source: Health Justice. 2014 Apr 1;2:7. doi: 10.1186/2194-7899-2-7 (PMC5151509; doi:10.1186/2194-7899-2-7)
Supplement: Supplementary file 3 — Authors’ original file for figure 3 [file 40352_2013_9_MOESM3_ESM.docx]

**Table 3.** Associations between current social support and history of trauma by type, with gender as a covariate.

|  | B | SE B | β |
| --- | --- | --- | --- |
| Any Trauma (n = 231) | -9.520 | 3.902 | -.159* |
| Gender = Female | 5.827 | 2.387 | .159* |
| Physical Trauma (n = 231) | -6.702 | 3.211 | -.137* |
| Gender = Female | 5.930 | 2.400 | .162* |
| Sexual Trauma (n = 230) | -4.906 | 2.387 | -.138* |
| Gender = Female | 8.228 | 2.460 | .225** |
| Crime-Related Trauma (n = 233) | -8.758 | 2.225 | -.248** |
| Gender = Female | 5.970 | 2.299 | .164** |

* p < .05

** p < .01
