# Supplementary material for: Associations between past trauma, current social support, and loneliness in incarcerated populations
Source: Health Justice. 2014 Apr 1;2:7. doi: 10.1186/2194-7899-2-7 (PMC5151509; doi:10.1186/2194-7899-2-7)
Supplement: Supplementary file 4 — Authors’ original file for figure 4 [file 40352_2013_9_MOESM4_ESM.docx]

**Table 4.** Associations between current social support and history of trauma by type and gender.

|  | Male (n = 83) | | | Female (n = 150^b^) | | |
| --- | --- | --- | --- | --- | --- | --- |
|  | B | SE B | β | B | SE B | β |
| Any trauma^a^ |  |  |  | -9.791 | 4.074 | -.195* |
| Physical trauma^a^ |  |  |  | -8.070 | 3.469 | -.189** |
| Sexual trauma | -.434 | 3.961 | -.012 | -7.626 | 2.979 | -.208* |
| Crime-related trauma | -6.867 | 3.940 | -.190 | -9.729 | 2.694 | -.285** |

* p < .05

** p < .01

1. Regressions predicting social support from any trauma and physical trauma were not conducted for men because only 2 men reporting not having experienced any trauma, and only 5 men reported not having experienced physical trauma.
2. For females, n = 147 (sexual trauma), 148 (physical trauma), 150 (crime-related trauma), 148 (any trauma).
