# Supplementary material for: Associations between past trauma, current social support, and loneliness in incarcerated populations
Source: Health Justice. 2014 Apr 1;2:7. doi: 10.1186/2194-7899-2-7 (PMC5151509; doi:10.1186/2194-7899-2-7)
Supplement: Supplementary file 5 — Authors’ original file for figure 5 [file 40352_2013_9_MOESM5_ESM.docx]

**Table 5.** Associations between current loneliness and history of trauma by type, with gender as a covariate (n=149).

|  | B | SE B | β |
| --- | --- | --- | --- |
| Any Trauma | 2.465 | 1.790 | .116 |
| Gender = Female | -1.373 | 1.017 | -.114 |
| Physical Trauma | 1.034 | 1.462 | .060 |
| Gender = Female | -1.536 | 1.024 | -.127 |
| Sexual Trauma | 2.822 | .994 | .235* |
| Gender = Female | -2.475 | 1.00 | -.205* |
| Crime-Related Trauma | 2.492 | .985 | .204* |
| Gender = Female | -1.417 | .977 | -.117 |

* p < .05

** p < .01
