# Supplementary material for: Associations between past trauma, current social support, and loneliness in incarcerated populations
Source: Health Justice. 2014 Apr 1;2:7. doi: 10.1186/2194-7899-2-7 (PMC5151509; doi:10.1186/2194-7899-2-7)
Supplement: Supplementary file 6 — Authors’ original file for figure 6 [file 40352_2013_9_MOESM6_ESM.docx]

**Table 6.** Associations between current loneliness and history of trauma by type and gender.

|  | Male (*n* = 83) | | | Female (*n* = 66) | | |
| --- | --- | --- | --- | --- | --- | --- |
|  | B | SE B | β | B | SE B | β |
| Any trauma^a^ |  |  |  | 1.909 | 2.043 | .116 |
| Physical trauma^a^ |  |  |  | .795 | 1.786 | .056 |
| Sexual trauma | 1.858 | 1.307 | .156 | 4.086 | 1.525 | .318* |
| Crime-related trauma | 2.535 | 1.310 | .210 | 2.443 | 1.503 | .199 |

* p < .05

** p < .01

1. Regressions predicting loneliness from any trauma and physical trauma were not conducted for men because only 2 men reporting not having experienced any trauma, and only 5 men reported not having experienced physical trauma.
